# Prospective genomic and epidemiologic surveillance of *Klebsiella pneumoniae* in a tertiary NICU

**DOI:** 10.1186/s13756-026-01753-4

**Published:** 2026-04-27

**Authors:** Carolin Böhne, Claas Baier, Jelena Erdmann, Ella Ebadi, Carina Zirkler, Marc Lindenberg, Dirk Schlüter, Sabine Pirr, Corinna Peter, Bettina Bohnhorst, Leonard Knegendorf

**Affiliations:** 1 Department of Pediatric Pneumology, Allergology and Neonatology, Hannover Medical School (MHH), Carl-Neuberg-Str. 1, 30625 Hannover, Germany; 2 Institute for Medical Microbiology and Hospital Epidemiology, Hannover Medical School (MHH), Carl-Neuberg-Str. 1, 30625 Hannover, Germany; 3 Infection Prevention and Control Department, AGAPLESION Diakonieklinikum Rotenburg, Elise-Averdieck-Str. 17, 27356 Rotenburg, Germany; 4 Institute for Molecular Bacteriology, TWINCORE, Centre for Experimental and Clinical Infection Research, a joint venture between the Helmholtz Centre for Infection Research and the Hannover Medical School, Feodor-Lynen-Str. 7, 30625 Hannover, Germany; 5 Cluster of Excellence RESIST (EXC 2155), Hannover Medical School (MHH), Carl-Neuberg Str. 1, 30625 Hannover, Germany

**Keywords:** *Klebsiella pneumoniae*, Neonatal intensive care unit, Colonization surveillance, Genomic epidemiology, Nosocomial transmission, Whole-genome sequencing, Very low birth weight, Machine learning, Macroclimate

## Abstract

**Background:**

*Klebsiella pneumoniae* complex (Kp) is a relevant neonatal pathogen colonizing preterm infants. While outbreak investigations often focus on multidrug-resistant strains, the epidemiology and genomic dynamics of wild-type Kp in nonoutbreak neonatal intensive care unit (NICU) settings remain elusive.

**Methods:**

We conducted a 30-month (October 2021 to March 2024) cohort study with weekly active, unselective colonization surveillance of all NICU patients to identify risk factors for nosocomial Kp acquisition and drivers of transmission in a tertiary 21-bed NICU/intermediate care unit (IMC) in Germany.

**Results:**

Among 936 patients, 8.7% carried Kp, of which 70.4% were nosocomial. Very low birth weight (VLBW; < 1500 g) was the only independent risk factor for nosocomial acquisition (adjusted odds ratio [aOR], 3.42; 95% CI, 1.29–9.32). Kp infections occurred in three Kp carriers (3.7%). Genomic analyses of at least the first isolate per patient (83 in total) revealed an oligoclonal population structure, with ten distinct sequence types (STs) underlying temporally overlapping clusters. Ten genomic clusters (median size, four patients) were identified, with markedly higher odds in VLBW infants (aOR, 8.76; 95% CI, 2.45–34.16). Nosocomial cluster-assigned cases had higher rates and longer durations of noninvasive ventilation and peripheral venous catheter use. Cluster prevalence showed climate-associated variation, with a six-feature extreme gradient boosting (XGBoost) model identifying temperature and humidity among the strongest predictors.

**Conclusions:**

Patient- and climate-associated parameters are main drivers of nosocomial wild-type Kp acquisition and cluster occurrence. Comprehensive surveillance and risk-adapted infection prevention and control support sustainable Kp control in VLBW infants.

**Supplementary Information:**

The online version contains supplementary material available at 10.1186/s13756-026-01753-4.

## Background

The Gram-negative *Klebsiella pneumoniae* complex (Kp) is known for colonizing preterm infants in neonatal intensive care units (NICUs) [1] and is one of the pathogens frequently leading to neonatal infections [2, 3]. Besides patient-related factors and the use of invasive devices and procedures [4–6], infection susceptibility is further influenced by pathogen-related attributes such as hypervirulent Kp isolates (hvKp) [7]. A gene-based grading system (Kleborate) [8] and a hypermucoviscous Kp phenotype (positive string test [9] or tellurite resistance [10]), may indicate increased virulence. Hypervirulence may also coincide with antibiotic resistance, including Carbapenem resistance [11].

Kp can persist in the environment (e.g. surfaces, medical products), thus being easily transmittable in NICUs [12, 13] and resulting in several outbreak reports from NICUs worldwide [13–16], including Carbapenem-resistant Kp [17, 18]. Since neonatal infections still represent a major cause of neonatal morbidity and mortality even in western, industrialized countries [19], close epidemiologic and molecular Kp monitoring in NICUs seems crucial from a clinical and infection prevention and control (IPC) perspective. In Germany, weekly microbiological colonization screening is mandatory in NICUs, following national recommendations of the Commission for Infection Prevention and Hygiene in Healthcare and Nursing (KRINKO) [20, 21]. To clarify transmission pathways, whole-genome sequencing (WGS) of Kp [22], including typing methods such as core genome multi-locus sequence typing (cgMLST) [23, 24], has been increasingly used. However, data on Kp transmission and infection apart from outbreak scenarios are still scarce.

Therefore, the objective of our study was to identify patient-related risk factors for nosocomial Kp acquisition in a non-outbreak, tertiary NICU/intermediate care (IMC) setting. The integration of epidemiological Kp surveillance and prospective IPC measures with a routine phenotypic and genomic analysis of Kp isolates, irrespective of resistance profile or outbreak suspicion, enabled us to assess Kp cluster prevalence. Furthermore, we contextualized the genomic features of Kp isolates with public genomes and explored the associations of Kp cluster prevalence with intensive care level and climate conditions using interpretable machine learning.

This comprehensive approach aimed to improve our understanding of the pathogen-patient-environment interactions as a basis for strengthening IPC strategies in the future.

## Methods

### Study type

We conducted a retrospective cohort study of our prospective surveillance program including all patients admitted to the tertiary 21-bed NICU/IMC of Hannover Medical School (MHH) from October 1, 2021, to March 31, 2024. The primary outcome was nosocomial Kp acquisition, defined as detection of Kp on or after day 3 of NICU/IMC stay. The secondary outcome was the prevalence of Kp clusters defined by genomic sequencing. Exploratory analyses compared nosocomial versus community-acquired cases and cluster-assigned versus non-cluster-assigned nosocomial cases. In addition, we characterized phenotypic and genomic features of all Kp isolates. The study adhered to the Strengthening the Reporting of Observational Studies in Epidemiology (STROBE) reporting guideline [25]. The ward setting, definitions, and data acquisition are detailed in the Additional Methods (see Additional file 1, Additional Methods).

In brief, a patient hospital stay (PHS) was defined as continuous inpatient episode on the NICU/IMC. The time at risk was defined as the number of days from admission to first detection of Kp in any microbiological sample.

### IPC concept and surveillance

Detailed information on our IPC concept, Kp surveillance program, and microbiological and molecular diagnostics are available in the Additional Methods (see Additional file 1, Additional Methods). In short, following microbiological admission screening, all NICU patients and IMC patients with an actual weight < 1500 g received weekly colonization screening of respiratory secretions and rectal swabs according to KRINKO [20, 21]. Microbiological diagnostics were conducted at the ISO 15189-accredited microbiology laboratory of MHH, and each newly emerged Kp isolate was sequenced using Illumina short-read technology.

### Statistical analysis and machine learning

To reduce immortal-time bias, the *at-risk* population for nosocomial Kp acquisition was defined as PHSs lasting ≥ 48 h with negative Kp status until that time, resulting in a reduced sample for multivariable regression analyses compared to the full cohort. We performed multivariable logistic regression to estimate adjusted odds ratios (aOR) with 95% profile-likelihood confidence intervals (CIs) for nosocomial acquisition, including gestational age (GA), very low birth weight (< 1500 g, VLBW, categorical), and sex as covariates (reference categories: absence of VLBW, female sex). Screening effort (number of surveillance samples per patient divided by the individual time at risk) was also included as a covariate to account for potential detection bias. A multivariable Cox proportional hazards model was additionally applied to account for time-dependent effects, with time at risk defined as days from admission to Kp acquisition or discharge (censoring). Covariates were identical to those used in the logistic regression model. Adjusted Hazard ratios (aHRs) with 95% profile-likelihood confidence intervals were estimated. An α level of 0.05 was used. Epidemiological and clinical variables were summarized as median (interquartile range [IQR]) or number (percentage). Group differences were tested using the chi-square test for categorical and Wilcoxon rank-sum test for continuous variables, with significance at *P* < 0.05. GraphPad Prism (version 10.2.0; GraphPad Software, Massachusetts, USA) was used for statistical analyses. Reporting of machine learning models followed the TRIPOD + AI statement [26]. Detailed information on machine learning analyses is available in the Additional Methods (see Additional file 1, Additional Methods). Machine learning analyses modeled cluster prevalence as the outcome in relation to patient-care metrics and climate factors including 127 weekly observations, with 27 weeks contributing to the test set. Hyperparameters were tuned to optimize model fit and interpretability, and models were used in a hypothesis-generating rather than predictive capacity. Model hyperparameters are detailed in Additional Table 1.

**Table 1:** Epidemiological and clinical characteristics of all PHSs stratified by Kp status and molecular cluster assignment

Parameter	All PHSs	PHSs without Kp	PHSs with any Kp	PHSs with nosocomial Kp	PHSs with nosocomial, cluster-assigned Kp
Total PHSs, n (^a^)	1009 (936)	927 (859^b^)	82 (81)	58 (57)	37 (37)
Length of PHSs, median [IQR], days	6.0 [2.4–15.2]	5.3 [2.2–13.6]	28.6 [9.1–50.7]	32.8 [17.1–62.4]	35.0 [26.2–57.4]
Time at risk^c^, median [IQR], days	6.0 [2.4–15.2]	5.3 [2.2–13.6]	7.0 [2.0–18.0]	12.5 [6.8–25.5]	16.0 [9.0–30.0]
Sex, male, n (%)	523/936 (55.9)	479/859 (55.8)	44/81 (54.3)	32/57 (56.1)	23/37 (62.2)
VLBW, n (%)	202/936 (21.6)	158/859 (18.4)	45/81 (55.6)	38/57 (66.7)	30/37 (81.1)
GA, median [IQR], days (weeks)	254 (36.3) [226 (32.3)–273 (39.0)]	256 (36.6) [230 (32.9)–274 (39.1)]	219 (31.3) [190 (27.1)–249 (35.6)]	208 (29.7) [188 (26.9)–237 (33.9)]	202 (28.9) [187 (26.7)–221 (31.6)]
Birth weight, median [IQR], grams	2503 [1610–3286]	2600 [1718–3340]	1470 [960–2225]	1300 [955–1878]	1220 [930–1478]

PHS, patient hospital stay; defined as a continuous inpatient episode on the NICU/IMC. Discharge or transfer ended the PHS, readmission started a new PHS. Kp, *Klebsiella pneumoniae* complex; A Kp case was any PHS with ≥ 1 Kp-positive specimen. Nosocomial Kp carriage was defined as acquisition on or after day three or later. VLBW, very low birth weight; GA, gestational age

^a^Numbers in parentheses indicate corresponding patients. ^b^Four patients acquired Kp during a later PHS. ^c^Time at risk is defined as length of stay in the NICU/IMC until Kp acquisition for positive cases or until discharge for negative cases

## Results

### Epidemiology and primary outcome

During the study period, 936 individual patients (median: 51 patients/month) were admitted to the NICU/IMC, generating 1009 PHSs and 14 684 patient days (median PHS length, 6 days) (Table 1). Microbiological diagnostics identified 81 Kp patients (prevalence, 8.7/100 patients; incidence density, 5.6 PHS/1000 patient days). Numbers differ slightly between patients, PHSs, and isolates due to multiple acquisitions in single patients (details in Table footnotes).

The mean (IQR) birth weight of all 936 NICU/IMC patients was 2503 (1610–3286) g, the mean (IQR) GA was 36.3 (32.3–39.0) weeks. VLBW infants accounted for 21.6% of all patients (202/936) and were threefold more frequent in the Kp subgroup. Among Kp patients, 70.4% (57/81) had nosocomial acquisition, of whom 66.7% (38/57) were VLBW infants. In multivariable logistic regression, VLBW was the only strong risk factor for nosocomial Kp acquisition (aOR, 3.42; 95% CI, 1.29–9.32), whereas higher gestational age was weakly protective (aOR, 0.98; 95% CI, 0.97–0.9986). After adjustment for screening effort, which was itself strongly associated with Kp acquisition, the association of VLBW remained robust (Table 2). In time-to-event analysis accounting for varying time at risk, VLBW infants showed a significantly increased hazard of nosocomial acquisition (aHR 3.49, 95% CI, 1.25–10.08). Exploratory analyses showed that nosocomial cases, compared with community-acquired cases, had a significantly higher proportion of VLBW infants, lower GA, and lower birth weight (Additional Table 2). During the time at risk, nosocomial cases also showed a high proportion of peripheral venous catheters (96.6%), noninvasive ventilation (75.9%), and intravenous antibiotic therapy (43.1%; Additional Table 2).

**Table 2:** Multivariable logistic and Cox proportional hazards regression analyses of nosocomial Kp or nosocomial Kp with molecular cluster assignment

Parameter	Adjusted OR [95% CI]^a^, nosocomial Kp	Adjusted HR [95% CI]^b^, nosocomial Kp	Adjusted OR [95% CI]^a^, nosocomial and cluster-assigned Kp	Adjusted HR [95% CI]^b^, nosocomial and cluster-assigned Kp
Sex	0.84 [0.47–1.50]	0.68 [0.39–1.16]	0.60 [0.29–1.23]	0.51 [0.25–1.02]
VLBW	**3.42 [1.29–9.32]**	**3.49 [1.25–10.08]**	**8.76 [2.45–34.16]**	**10.78 [2.61–49.69]**
GA	**0.98 [0.97–0.9986]**	1.01 [0.99–1.02]	0.99 [0.97–1.01]	1.01 [0.99–1.03]
Screening effort^c^	**4.83 [2.01–11.10]**	**15.26 [7.64–29.11]**	**3.62 [1.05–10.78]**	**21.03 [7.63–53.59]**

Kp, *Klebsiella pneumoniae* complex; The *at-risk population* analyzed in the regression models comprised patients hospitalized for ≥ 48 h and Kp negative until that time point (landmark definition; n = 659 Kp negative, n = 57 nosocomial Kp, and n = 37 nosocomial cluster-assigned patients). VLBW, very low birth weight; GA, gestational age

^a^Adjusted odds ratios with 95% profile-likelihood confidence intervals are from multivariable logistic regression at the patient level, comparing nosocomial Kp acquisition or nosocomial Kp with molecular cluster assignment against no Kp detection. Because of collinearity, VLBW (< 1500 g birth weight) and not birth weight was retained in the multivariable model. ^b^Adjusted hazard ratios with 95% profile-likelihood confidence intervals are from Cox proportional hazards regression based on the same dataset, with individual time at risk, i.e., length of stay in the NICU/IMC until Kp acquisition for positive cases or until discharge for negative cases, as time variable. Kp acquisition was defined as event and discharge without Kp as censor. Reference categories for both analyses: male sex and absence of VLBW. ^c^Screening effort was defined as the number of surveillance samples per patient divided by the individual time at risk. Inclusion of screening effort did not materially change the association of VLBW with Kp acquisition in the logistic regression model. Bold values indicate statistical significance (α = 0.05; *P* < 0.05)

Three patients (3.7%) developed a Kp infection (Table 3). One patient developed infection postoperatively following colonization acquired at a transferring hospital, whereas in the two other patients the infection occurred during prolonged hospitalizations; both were profoundly immunocompromised, including one solid organ transplant recipient.

**Table 3:** Epidemiological and clinical characteristics of Kp infections

Epidemiological parameters	Patient A	Patient B	Patient C
GA, category	Early preterm	Early preterm	Term
Birth weight, grams	[1500–2499]	[1500–2499]	[2500–4200]
Time at risk^a^, days	0^b^	48	56
Time until infection, days	5	48	56
Length of stay, days	11	86	85
*Clinical parameters during time until infection*			
Central venous catheter, days	5	33	56
Peripheral venous catheter, days	5	22	5
Invasive ventilation, days	1	14	56
Non-invasive ventilation, days	1	11	0
Transurethral catheter, days	0	9	10
Surgery	yes	no	yes

GA, gestational age; Kp, *Klebsiella pneumoniae* complex; Kp infection was defined as the presence of Kp in a diagnostic microbiological specimen (e.g. blood culture), combined with ≥ 5 days of antibiotic treatment and clinical documentation

^a^Time at risk refers to days from admission until acquisition of the Kp isolate. ^b^Postoperative infection following Kp colonization at a transferring hospital

### Nosocomially acquired Kp and molecular cluster assignment

Eighty-three isolates from 79 patients were available for molecular analysis. 40 patients (40/81 [49.4%]) were assigned to 10 genomic clusters, each comprising two to ten patients. Ten distinct sequence types (STs) underpinned the clusters, accounting for 44/83 (53.0%) of isolates. Cluster assignment was more frequent among nosocomial Kp patients (37/57 [64.9%]) than among community-acquired Kp patients (3/24 [12.5%]). In multivariable analysis, VLBW was associated with substantially higher odds of cluster assignment (aOR, 8.76; 95% CI, 2.45–34.16; Table 2).

Exploratory subgroup analyses showed that nosocomial cluster-assigned cases had more often a VLBW and had higher rates of noninvasive ventilation compared with non-cluster assigned cases (Table 4). Moreover, the duration of noninvasive ventilation and peripheral venous catheterization was significantly longer in cluster-assigned cases (Additional Table 3).

**Table 4:** Epidemiological and clinical parameters of nosocomial Kp PHSs stratified by molecular cluster assignment

Parameter	All nosocomial Kp PHSs (A)	Nosocomial Kp PHSs with molecular cluster assignment (B)	Nosocomial Kp PHSs without molecular cluster assignment (C)	*P*-value^c^ (B vs. C)
*Epidemiological parameters*	**n = 58**	**n = 37**	**n = 21**	
Total PHSs, n (^a^) [% of all PHSs (A)]	58 (57)^b^	37 (37) [63.8%]	21 (21) [36.2%]	–
Length of PHSs, median [IQR], days	32.8 [17.1–62.4]	35.0 [26.2–57.4]	24.7 [12.1–67.9]	.27
Time at risk of PHSs, median [IQR], days	12.5 [6.8–25.5]	16.0 [9.0–30.0]	7.0 [4.0–21.5]	0.06
*Patient associated parameters*	**n = 57**	**n = 37**	**n = 21**	
VLBW, n (%)	38 (66.7)	30 (81.1)	8 (38.1)	**0.0009**
GA, median [IQR], days (weeks)	208 (29.7) [188 (26.9)–237 (33.9)]	202 (28.9) [187 (26.7)–221 (31.6)]	238 (34.0) [189.5 (27.1)–260.5 (37.2)]	**0.02**
Birth weight, median [IQR], grams	1300 [955–1878]	1220 [930–1478]	1670 [985–2952]	**0.01**
Sex, male, n (%)	32 (56.1)	23 (62.2)	10 (47.6)	0.28
Death, n (%)	6 (10.5)	6 (16.2)	0 (0)	0.05
*Clinical parameters per PHS*	**n = 58**	**n = 37**	**n = 21**	
Central venous catheter, n (%)	25 (43.1)	15 (40.5)	10 (47.6)	0.60
Peripheral venous catheter, n (%)	56 (96.5)	37 (100)	19 (90.5)	0.06
Invasive ventilation, n (%)	23 (39.7)	15 (40.5)	8 (38.1)	0.85
Non-invasive ventilation, n (%)	44 (75.9)	32 (86.5)	12 (57.1)	**0.01**
Transurethral catheter,n (%)	10 (17.2)	4 (10.8)	6 (28.6)	0.08
Surgery, n (%)	8 (13.8)	5 (13.5)	3 (14.3)	0.93
Systemic (intravenous) antibiotic therapy, n (%)	25 (43.1)	16 (43.2)	9 (42.9)	0.97
Cefotaxime, n (%)	11 (18.9)	8 (21.6)	3 (14.3)	0.49
Vancomycin, n (%)	8 (13.8)	3 (8.1)	5 (23.8)	0.09
Meropenem, n (%)	9 (15.5)	6 (16.2)	3 (14.3)	0.84
Tobramycin, n (%)	22 (37.9)	17 (45.9)	5 (23.8)	0.09

PHS, patient hospital stay; Kp, *Klebsiella pneumoniae* complex; VLBW, very low birth weight; GA, gestational age

^a^Corresponding number of single patients. ^b^One patient initially carried a non-cluster-assigned Kp strain and, after discharge and subsequent readmission to the neonatal intensive care unit (NICU) during the same overall hospital stay, acquired a cluster-assigned Kp strain. This resulted in two separate PHSs for that patient, one counted in column (B) and one in column (C). Consequently, the number of single *patients* in column (A) totals 57, whereas the sum of patients in columns (B) and (C) equals 58, consistent with the total number of PHSs (A). ^c^Chi-square test for categorical parameters and Wilcoxon rank sum test for continuous parameters, respectively. Bold values indicate statistical significance (*P* < 0.05)

### Integrated phenotypic and genomic analysis

**Fig. 1:**
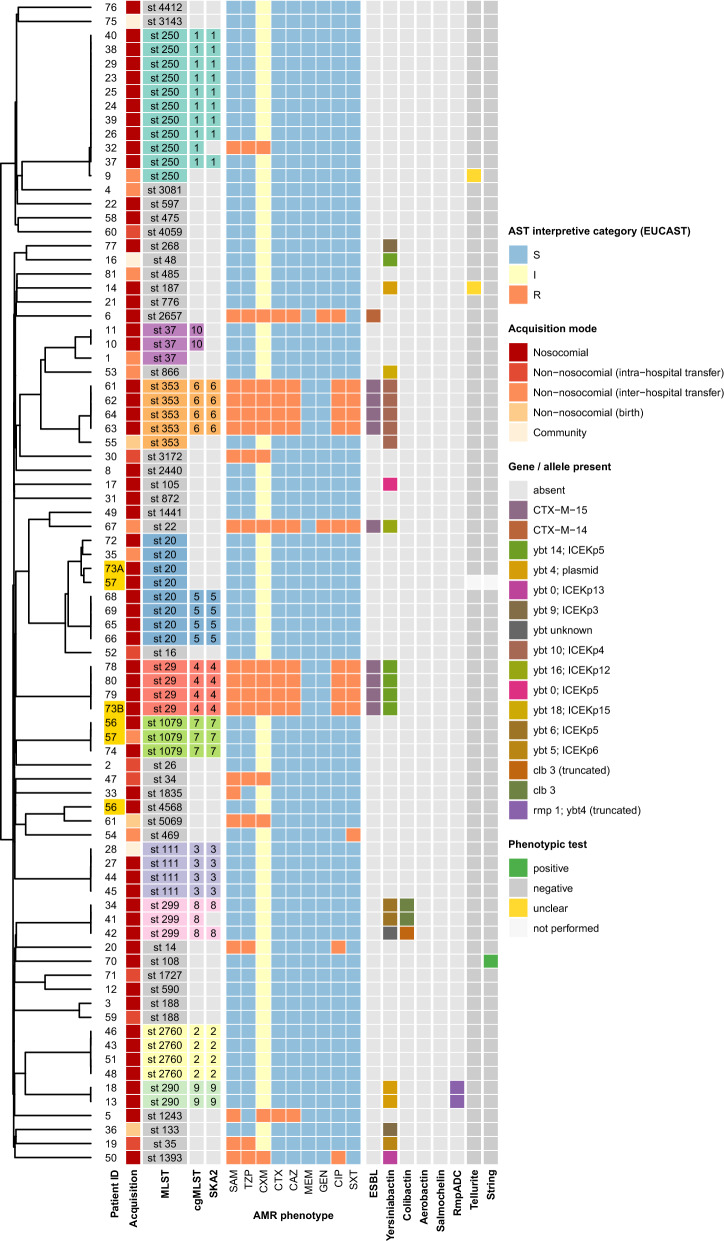
Phylogeny, antimicrobial susceptibility, and genomic/phenotypic features of 83 Kp isolates. Rows represent individual isolates ordered by a midpoint-rooted neighbor-joining dendrogram based on core-genome multilocus sequence typing (cgMLST) allele distances calculated in Ridom SeqSphere + (left). The central heatmap displays EUCAST antimicrobial susceptibility interpretations (susceptible [S], increased exposure [I], resistant [R]) for nine agents: ampicillin/sulbactam (SAM), piperacillin/tazobactam (TZP), cefuroxime (CXM), cefotaxime (CTX), ceftazidime (CAZ), meropenem (MEM), gentamicin (GEN), ciprofloxacin (CIP), and trimethoprim/sulfamethoxazole (SXT). Left annotations: patient ID (duplicates highlighted), acquisition mode (darkest = nosocomial; lightest = community), and cluster assignment by MLST, cgMLST, and SKA2 (shared color key); unclustered (“singleton”) entries are shown in light gray. Right annotations: presence of extended-spectrum β-lactamase (ESBL) loci and major virulence determinants (Yersiniabactin, Colibactin, Aerobactin, Salmochelin, RmpADC; colored by allele). Phenotypic results for tellurite resistance and string test shown as positive, negative, or unclear; light gray indicates tests not performed

Phenotypic hypervirulence markers yielded one positive string test and two indeterminate tellurite results; none belonged to a genomic cluster or caused infection. Kleborate analysis did not identify hypervirulent strains. Three isolates within cgMLST cluster 8 carried yersiniabactin and colibactin, but none caused infection (Additional File 2, Additional Table 3). Most isolates (63/83) exhibited a wild-type antimicrobial resistance profile; 10 had an extended-spectrum β-lactamase (ESBL) phenotype, of which eight belonged to clusters (ST29 and ST353, four isolates each, all blaCTX-M-15-positive; Fig. 1). No carbapenem resistance was detected. All cgMLST clusters displayed consistent phenotypic profiles, with a single discrepant isolate in cluster 1 (Split k-mer analysis [SKA] distance > 20 single nucleotide polymorphisms [SNPs]). This isolate was retained within the cgMLST-defined cluster for IPC purposes during prospective surveillance.

SNP comparison against public genomes (Bacterial and Viral Bioinformatics Resource Center [BV-BRC] database) [27] revealed the closest match (48 SNPs) to a marine isolate from Norway [28]; this strain (ST866) was identified upon admission in a patient residing at the German North Sea coast (Additional File 2, Additional Table 5).

Throughout the study, molecular typing results were presented during regular interdisciplinary IPC audits. The median time from sampling to completed WGS was 3.1 weeks (IQR, 2.6–4.6).

### Association of climate and patient-care factors with molecular cluster prevalence

Kp cluster prevalence showed seasonal variations (Additional Fig. 1). To examine external drivers, machine learning models were trained with climate variables and nurse-to-patient ratios, including 4-week lagged values based on exploratory analyses suggesting temporal associations. Model performance was evaluated using a season-stratified 80:20 split. The reported R^2^ reflects out-of-sample performance on the held-out test set and quantifies the proportion of variance explained within this specific temporal dataset.

**Fig. 2:**
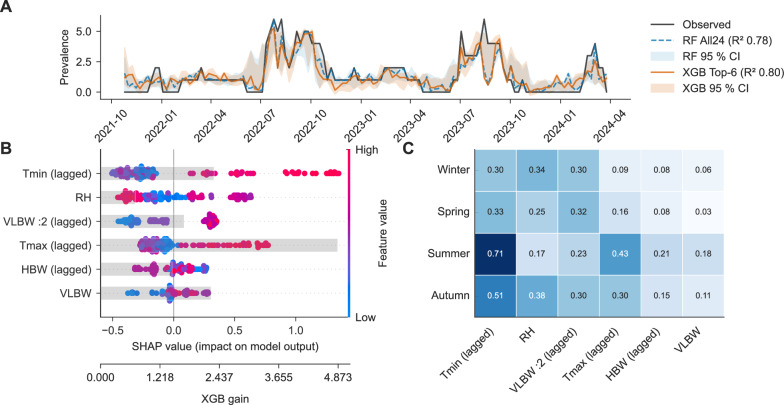
Machine learning-based prediction of molecular cluster prevalence and model interpretation. **A** weekly observed cluster prevalence (black line) vs predicted prevalence from Random Forest (blue dotted line) and reduced-feature extreme gradient boosting (XGBoost) model (orange line). Shaded bands indicate pointwise 95% bootstrap intervals obtained by refitting each model on 800 bootstrap resamples of the weekly dataset and predicting across all weeks. R^2^ values refer to performance on a single within-season 80:20 train-test split. **B** Global feature importance in the reduced-feature XGBoost model. Shapley Additive Explanations (SHAP) beeswarm plot shows mean absolute SHAP values (ranked by importance) and individual SHAP values per observation (colored by feature value). Grey bars indicate gain-based feature importance. **C** Seasonal variation in feature importance. Heatmap of mean absolute SHAP values stratified by meteorological season. *Abbreviations* [Tmin (lagged)] minimum outdoor temperature, 4-week lag; [RH] current humidity; [VLBW:2 (lagged)] very low birth weight (< 1500 g) patients treated with a 1:2 nurse-to-patient ratio (1 nurse per 2 patients), 4-week lag; [Tmax (lagged)] maximum outdoor temperature, 4-week lag; [HBW (lagged)] peak number of patients with birth weight > 1500 g (“higher birth weight”) treated, 4-week lag; [VLBW] current peak number of very low birth weight patients treated (all nursing ratios). For definitions, see Additional Methods

Linear models (elastic net, lasso, and ridge) showed limited predictive ability (Additional Table 1). An extreme gradient boosting (XGBoost) model using a reduced feature set provided the best fit to the observed variance (hold-out R^2^ = 0.80). A random forest model achieved a comparable fit (hold-out R^2^ = 0.78) but required inclusion of 24 features (Fig. 2A; full feature set in Additional file 1, Additional Methods). The largest discrepancy between observed and predicted prevalence occurred in August 2023, mainly attributable to cluster 5 (ST20).

Feature importance was evaluated using gain-based metrics and Shapley Additive Explanations (SHAP). A higher minimum outdoor temperature four weeks earlier (lagged) consistently emerged as the top feature across seasons—weeks with little or no cooling phases (warmer nights) were followed by higher cluster prevalence, while the direct effect on indoor environmental conditions was not measured. Humidity showed a complex, bidirectional influence on predicted outcomes across its observed range and exerted stronger relative effects in winter and autumn, while the VLBW nurse-to-patient ratio, correlated with higher predicted prevalence, contributed more modestly but stably over time (Fig. 2B and C, Additional Fig. 2). Learning curves demonstrated that cross-validated R^2^ values remained below 0.5 across increasing training fractions, indicating moderate generalizability (Additional Fig. 3).

## Discussion

In this study, we comprehensively analyzed epidemiological and genomic Kp surveillance at a tertiary NICU/IMC in a non-outbreak setting, including an assessment of risk factors for nosocomial Kp acquisition and an evaluation of cluster prevalence using machine learning.

Kp was detected in ~ 8–9% of hospitalized patients, consistent with 2016–2018 data from our unit [29], and about ten times higher than local MRSA prevalence [30]. In contrast, intestinal Kp carriage was 40–60% in Indian VLBW preterm infants [31], while adult intensive care unit (ICU) colonization rates ranged between 6 and 20% [32, 33]. More than two thirds of Kp cases in our NICU were nosocomial, consistent with studies showing that the initial microbiome in preterm infants is shaped by the unit’s environment, including other infants, surfaces, staff and parents [34–36]. Although VLBW patients accounted for only ~ 21% of all PHSs, VLBW was the only independent factor for nosocomial acquisition, with 67% of nosocomial cases and 81% of cluster-associated PHSs occurring in VLBW infants. Time-to-event analyses supported that the association was not solely explained by longer time at risk. Nevertheless, residual confounding by care intensity cannot be fully excluded, and we cannot conclusively distinguish between increased biological susceptibility and increased exposure associated with intensified medical management in VLBW infants.

Kp is a leading cause of neonatal sepsis following intestinal colonization [37–39]. Known infection risk factors include patient-related factors, invasive procedures, and prior antibiotic use [40], although pathomechanisms remain elusive [32]. We observed increased rates and durations of noninvasive ventilation and peripheral venous catheter use in our nosocomial cluster-associated patients. Peripheral catheters are a recognized risk factor for nosocomial bloodstream infections in VLBW patients [41] and have been addressed in German IPC recommendations [20]. However, given the longer time at risk in nosocomial patients, these findings should be interpreted cautiously, as they may reflect opportunity bias.

Most NICU Kp reports focus on multidrug-resistant or hypervirulent strains during outbreaks [12–15, 17]. In contrast, our colonization screening explicitly addresses also the carriage of susceptible (wild-type) Kp isolates. No hypervirulent or carbapenem-resistant isolates were identified, suggesting that classical Kp remains the predominant colonizer in NICUs [15, 42, 43]. Although phenotypic markers such as string test and tellurite resistance may yield false positives, their use enabled real-time triaging and IPC adjustments before sequencing results were available.

ESBL production was rare and largely confined to two clusters (ST29 and ST353), both harboring blaCTX-M-15, consistent with sporadic import rather than endemic persistence. High genomic consistency within clusters—observed with both cgMLST and SKA in this study supports cgMLST-based IPC interventions. However, the identification of one isolate with divergent resistance within a cgMLST-defined cluster (SNP distance > 20) underscores the value of SNP-level analysis to refine cluster boundaries [44]. In addition, the literature indicates that, particularly for taxonomically complex species such as *Enterobacter cloacae*, reliance on cgMLST-based clustering alone may lead to misinterpretation [45]. In our prospective IPC workflow, such isolates were retained within cgMLST-defined clusters to prioritize sensitivity for transmission detection. In summary, for the operational use of genomic data in the IPC context, we consider it advisable to interpret cgMLST-based cluster assignments in close conjunction with the epidemiological context and, particularly in cases of uncertainty, to complement them with higher-resolution analytical approaches such as SKA. Interestingly, the closest genomic match for one isolate (ST866) was a marine environmental strain from Norway, illustrating potential environmental influx and the value of contextualizing local isolates against public datasets.

While the spread of hospital-associated, carbapenem-resistant lineages of Kp is well-documented [46], we found no clusters involving globally circulating lineages. Consistent with the absence of hvKp/carbapenem resistance and rapid containment, the infection attack rate of 3.7% of all Kp patients (0.3% of admissions) was comparable to prior reports [6, 39].

Kp was repeatedly introduced and also transmitted, but cluster size remained small (median, four patients), suggesting that transmission was usually limited in size and duration, consistent with timely IPC interventions. These included training/feedback, cohorting/isolation, encouraging hand hygiene, intensified patient and environmental screening, and enhanced disinfection [12–15, 17]. Audit-style feedback on epidemiological and genomic findings was progressively strengthened during the study, reaching near-weekly intervals, with rising interdisciplinary participation. Similar formats have proven effective in teaching IPC [47]. Clusters detected by molecular typing were addressed systematically during audits and in scheduled or ad hoc interdisciplinary training sessions, prompting targeted interventions to interrupt transmission chains and prevent establishment of dominant ‘ward clones’.

Six pairs of twins were included among cluster patients, highlighting elevated risks for patient-to-patient or parent-to-patient transmission [35, 36]. Though skin-to-skin (kangaroo) care in VLBW infants facilitates bacterial exchange, it remains a standard practice that favors neurodevelopment and microbiome stability [48, 49]. From an IPC perspective, awareness of this potential transmission route in preterm multiples is essential.

Seasonal variation in Kp cluster prevalence, despite stable NICU occupancy, prompted further investigation using machine learning models. Tree-based models, particularly a reduced-feature XGBoost model, explained up to 80% of variance in weekly prevalence within the observed dataset, while linear models failed to capture nonlinear relationships. These findings suggest that Kp transmission in NICUs is shaped not only by patient-specific factors, but also by structural and environmental variables with delayed effects.

A higher minimum daily outdoor temperature four weeks earlier was the most influential predictor, consistent with multicenter studies linking temperature with bloodstream infection incidence and environmental Kp abundance [50–53]. In particular, Schwab et al. reported that nosocomial bloodstream infection rates in German ICUs were approximately 16% higher in months with mean temperatures ≥  20 °C compared with <  5 °C, with the strongest association observed for temperatures in the preceding month [50]. Additional drivers in our data included relative humidity and increased intensive-care density/capacity, with the VLBW nurse-to-patient ratio serving as a surrogate for patient-related risk. We hypothesize that outdoor climate conditions may influence staff and visitor colonization on one hand and accumulation of Kp in the ward environment (e.g., surfaces, devices, sinks, wastewater systems, or other moist reservoirs) on the other hand. The importance of lagged features appears biologically and operationally plausible. The lag of four weeks could reflect gradual amplification in environmental burden followed by increased exposure risk and subsequent transmission within the ward setting. The lag may also correspond to the interval between patient acquisition event and eventual microbiological detection. This interval could encompass asymptomatic colonization, intra-ward spread, and eventual sampling. However, because our analysis relied on outdoor rather than indoor environmental measurements, these mechanisms remain inferential.

These models were exploratory and used to quantify associations, not to predict future risk. The identified associations between macroclimatic variables and cluster prevalence should not be interpreted as direct mechanistic effects but rather as statistical relationships within the observed dataset that warrant further investigation in larger multicenter cohorts incorporating environmental measurements. Moreover, the process of variable/feature selection probably influenced model performance and effect estimates, particularly in our study with a limited number of temporal observations. Nonetheless, the models offer a framework for hypothesis generation and provide actionable insights for IPC planning, such as anticipating periods of increased transmission risk. Nonlinear relationships, such as the bidirectional effect of humidity, underline the complexity of these dynamics. Expansion of an ST20 cluster despite predictively unfavorable external influences may suggest clone-specific transmissibility, although we could not find literature specifically supporting this.

This study has several limitations that should be considered when interpreting the findings. This single-center retrospective cohort study includes weekly colonization screening mandated by German IPC regulations, which may increase awareness of bacterial carriage and antibiotic use in preterm infants. Therefore, it may not be directly generalizable to other NICU settings. Routine Kp sequencing was subject to a time lag, limiting its utility for immediate IPC interventions. While we adjusted the primary analysis to reduce immortal time bias, differences in observation periods between nosocomial and community-acquired cases may have introduced opportunity bias. The machine learning models were not externally validated, and prone to overfitting, requiring larger multicenter datasets for predictive use. The R^2^ estimate reported reflects model fit within 127 weekly observations of a single-center cohort and should be interpreted as descriptive within this specific temporal context rather than as evidence of robust predictive performance across settings. The study period was relatively short for drawing climate-related conclusions. In addition, the effect of daily outdoor temperature, rather than indoor temperature, on cluster prevalence was analyzed. Practical observations on the ward suggest that, despite the presence of ventilation systems, outdoor temperature affected indoor thermal conditions. Nonetheless, the absence of indoor environmental measurements further limits the ability to infer any causal pathways. In sum, the observed associations should therefore be interpreted as hypothesis-generating rather than confirmatory evidence of climate-driven transmission dynamics. Additional potential transmission vectors—such as staff and visitor carriage or environmental reservoirs—were not systematically assessed.

Because molecular results were delayed, findings mainly informed medium- to long-term IPC strategies in our setting. Streamlined workflows (e.g. usage of Oxford Nanopore Sequencing technologies [54, 55] or more rapid Illumina sequencing) may allow more actionable short-term interventions in the future. In response to these findings, we have initiated the implementation of Oxford Nanopore sequencing within our prospective surveillance framework and are optimizing laboratory workflows to reduce turnaround times for isolates undergoing WGS.

## Conclusions

In a tertiary NICU/IMC, prospective genomic surveillance within a structured IPC program showed that Kp transmission was mainly driven by wild-type isolates. Infection rates were uncommon and genomic clusters remained small, consistent with the effect of routine screening and sequencing-informed audits with rapid feedback into practice. VLBW infants were the main risk group for nosocomial acquisition and were disproportionately affected by cluster-associated Kp. Cluster prevalence reflected the combined influence of VLBW patient vulnerability, structural, and environmental influences, with higher rates observed following periods of sustained warmer nights. These findings outline a scalable surveillance-to-action approach that may help further limit transmission in highly vulnerable neonatal populations. The identified time lag suggests a planning horizon for risk-triggered IPC: timely staff refreshers, increased environmental disinfection, focused screening, and the preemptive reverse isolation of infants at highest risk during anticipated higher-risk periods could be measures worth evaluating their efficacy in future studies. 

## Supplementary Information

Additional file1 (PDF 499 KB)

Additional file2 (XLSX 105 KB)

## Data Availability

The patient data used in this study are confidential in accordance with the German Data Privacy Act, the ethics committee and the data protection commissioner of Hannover Medical School. Patient-related data such as date of ward admission, age, sex, underlying disease or length of stay are indirect identifiers that might enable patient identification. To protect patient confidentiality and participant privacy, the data used for this study can be obtained in anonymized and condensed form only, according to the Data Privacy Act. Interested researchers who meet the criteria for access to confidential data may contact the data protection commissioner of the Hannover Medical School (datenschutz@mh-hannover.de) and one of the corresponding authors (e.g., boehne.carolin@mh-hannover.de) to obtain access to anonymized data, as approved by the data protection commissioner and the ethics committee of the Hannover Medical School. All isolate-level metadata used in this study are provided in the Additional File 2 (Additional Tables 4, 5). Whole-genome sequencing data have been deposited in the European Nucleotide Archive under study accession PRJEB98673; individual sample accession numbers are listed in Additional Table 4. All isolate-level metadata used in this study are provided in the Additional File 2 (Additional Tables 4, 5). Whole-genome sequencing data have been deposited in the European Nucleotide Archive under study accession PRJEB98673; individual sample accession numbers are listed in Additional Table 4.

## Abbreviations

aHR: Adjusted Hazard ratio
aOR: Adjusted odds ratio
BV-BRC: Bacterial and Viral Bioinformatics Resource Center
cgMLST: Core genome multi-locus sequence typing
CI: Confidence interval
ESBL: Extended-spectrum β-lactamase
GA: Gestational age
hvKp: Hypervirulent *Klebsiella pneumoniae* complex
ICU: Intensive care unit
IMC: Intermediate care unit
IPC: Infection prevention and control
Kp: *Klebsiella pneumoniae* complex
KRINKO: Commission for infection prevention and hygiene in healthcare and nursing at the Robert Koch Institute, Germany (Kommission für Infektionsprävention in medizinischen Einrichtungen und in Einrichtungen und Unternehmen der Pflege und Eingliederungshilfe)
MHH: Hannover Medical School (Medizinische Hochschule Hannover)
MRSA: Methicillin-resistant Staphylococcus aureus
NICU: Neonatal intensive care unit
PHS: Patient hospital stay
SHAP: Shapley Additive Explanations
SKA: Split k-mer analysis
SNP: Single nucleotide polymorphism
ST: Sequence type
VLBW: Very low birth weight
VRE: Vancomycin-resistant enterococci
WGS: Whole genome sequencing
XGBoost: Extreme gradient boosting
